# Etymologia: Leprosy

**DOI:** 10.3201/eid2112.ET2112

**Published:** 2015-12

**Keywords:** leprosy, Hansen disease, Mycobacterium leprae, bacteria, Armauer Hansen, Albert Neisser

## Leprosy [lepʹrə-se]

From the Greek *lepros*, “scaly,” leprosy is a chronic infectious disease of man caused by *Mycobacterium leprae* and principally affects the peripheral nerves and skin (Figure). The earliest known skeletal evidence for leprosy has been found in India and dates to 2000 bce. This finding suggests that the first textual references to leprosy are in ancient Sanskrit hymns of the *Atharva Veda*. The armies of Alexander the Great may have brought leprosy from India to western Asia circa 326 bce, and it spread further west when Roman armies campaigning in Asia Minor and Syria returned home (62 bce). The Romans referred to leprosy as *elephantiasis graecorum* and could distinguish between the similar symptoms of lymphatic filariasis, or *elephantiasis arabum*.

**Figure:**
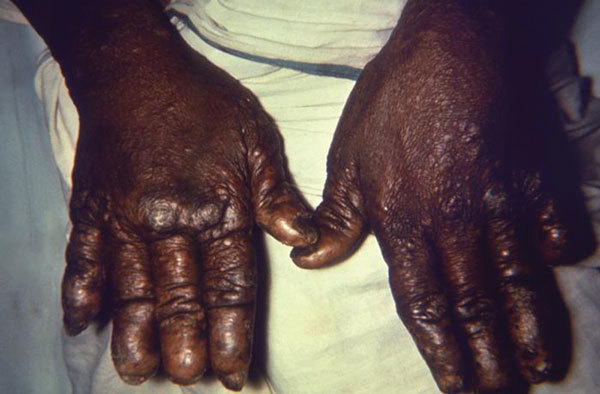
Dorsal surface of the hands of a patient with a case of nodular lepromatous leprosy, which under the newer World Health Organization (WHO) standards, is classified as multibacillary (MB), leprosy.

Norwegian physician Armauer Hansen identified the causative agent, *Mycobacterium leprae*, in 1873; however, it was successfully identified as a bacterium only in 1879 by a young German physician, Albert Neisser, who attempted to take credit for the discovery. Today, leprosy is also known as Hansen disease.
